# Programming ability prediction: Applying an attention-based convolutional neural network to functional near-infrared spectroscopy analyses of working memory

**DOI:** 10.3389/fnins.2022.1058609

**Published:** 2022-12-01

**Authors:** Xiang Guo, Yang Liu, Yuzhong Zhang, Chennan Wu

**Affiliations:** ^1^School of Information and Electronic Engineering, Zhejiang University of Science and Technology, Hangzhou, China; ^2^School of Electrical and Computer Engineering, University of Alberta, Edmonton, AB, Canada

**Keywords:** programming ability, fNIRS, working memory, convolutional neural network, attention mechanism

## Abstract

Although theoretical studies have suggested that working-memory capacity is crucial for academic achievement, few empirical studies have directly investigated the relationship between working-memory capacity and programming ability, and no direct neural evidence has been reported to support this relationship. The present study aimed to fill this gap in the literature. Using a between-subject design, 17 programming novices and 18 advanced students performed an n-back working-memory task. During the experiment, their prefrontal hemodynamic responses were measured using a 48-channel functional near-infrared spectroscopy (fNIRS) device. The results indicated that the advanced students had a higher working-memory capacity than the novice students, validating the relationship between programming ability and working memory. The analysis results also showed that the hemodynamic responses in the prefrontal cortex can be used to discriminate between novices and advanced students. Additionally, we utilized an attention-based convolutional neural network to analyze the spatial domains of the fNIRS signals and demonstrated that the left prefrontal cortex was more important than other brain regions for programming ability prediction. This result was consistent with the results of statistical analysis, which in turn improved the interpretability of neural networks.

## Introduction

In the past decade, computer science and programming have been applied in many fields, such as engineering, social sciences music, art, and biology (Buitrago Flórez et al., 2017). Consequently, programming ability has become a basic skill that students may need to master. Several studies have suggested that students with better programming ability have better problem-solving skills and logical reasoning ability (Tu and Johnson, 1990; Shute, 1995; Wing, 2008; Werner et al., 2012; Ivanova et al., 2020; Relkin et al., 2021).

Programming requires memorization of a wide range of information and the ability to manipulate the information at the same time. Students process and hold this information in their working memory, the mode of information storage in the human brain as proposed by cognitive psychology (Baddeley and Hitch, 1974). Working memory is used to store task-relevant information for further application in the process of performing cognitive tasks. It is a memory system with limited capacity for temporary processing and storage of information that supports human thought processes by providing an interface for perception, long-term memory, and action (Baddeley, 2003). Working memory is not only the core of human cognition but also an important component of learning, reasoning, problem-solving, and intellectual activity (Barrouillet and Lépine, 2005; Baddeley, 2010). Working memory plays a critical role in learning. Extensive research has demonstrated a significant relationship between working-memory capacity and academic achievement (Swanson and Alloway, 2012; Anmarkrud et al., 2019). Studies have shown that high performance in math and readings are linked to high working-memory performance (Purpura and Ganley, 2014; Cantin et al., 2016). However, to the best of our knowledge, none of the prior studies showed that programming ability was related to working memory. Nevertheless, since code comprehension involves diverse cognitive domains, including math, logic, and language (Ivanova et al., 2020), programming ability may also be assumed to be related to students’ working memory.

The n-back task is one of the most popular experimental paradigms for measuring working memory. The n-back paradigm is a continuous task paradigm (Cohen et al., 1997). In the n-back experiment, participants are asked to monitor a series of verbal/non-verbal stimuli and indicate whether the stimuli currently presented are the same as those that appeared in n trials previously (Braver et al., 1997). The traditional n-back experimental measurements include evaluation of accuracy and reaction time.

In recent years, many researchers have combined functional magnetic resonance imaging (fMRI), electroencephalography (EEG), and functional near-infrared spectroscopy (fNIRS) to measure physiological signals in task-evoked experimental processes to obtain the underlying neuroscientific mechanism of working memory (Ragland et al., 2002; Herff et al., 2013; Lv et al., 2014, 2015; Yeung et al., 2021).

In comparison with fMRI, fNIRS requires a small volume and is lightweight and portable while yielding images with a higher temporal resolution. fNIRS also shows a faster spatial response speed than EEG (Ferrari and Quaresima, 2012; Hong and Yaqub, 2019; Quaresima and Ferrari, 2019; Yang et al., 2019).

Functional near-infrared spectroscopy is a neuroimaging technique for measurement of hemodynamic processes in the brain. In this technique, the absorption of infrared light with a wavelength of 650–950 nm passing through the brain tissue is evaluated to monitor the changes in blood oxygen concentration in different brain tissue regions (Pinti et al., 2020) and obtain insights into the same activation patterns as fMRI. Matthes and Gross (1938) first demonstrated the spectroscopic determination of oxygenated hemoglobin (HbO) and deoxygenated hemoglobin (HbR) in human tissue in the red and near-infrared regions. In 1993, some research groups proved that fNIRS could be used to investigate brain activity non-invasively (Chance et al., 1993; Hoshi and Tamura, 1993; Villringer et al., 1993). Wolf et al. (2002) first used near-infrared spectroscopy and detected significant changes in the local concentrations of HbO and HbR during brain activity. When the brain executes a task, the increased metabolic demands for oxygen and glucose result in an oversupply of local cerebral blood flow (CBF) to satisfy the increased metabolic demand. CBF is regulated by several neurovascular coupling mechanisms. Therefore, the excessive supply of local CBF leads to an increase in HbO concentration and a decrease in HbR concentration (Pinti et al., 2020). Some previous studies have shown that fNIRS is sensitive to load-dependent working-memory changes in activation (Herff et al., 2013; Meidenbauer et al., 2021) and have demonstrated linear increments in HbO concentrations in frontal activation based on n-back levels (Ayaz et al., 2012; Yeung et al., 2021). The results of a meta-analysis of brain imaging data acquired during the n-back task showed that the participants’ prefrontal cortex was activated consistently (Nystrom et al., 2000; Owen et al., 2005). Therefore, in this study, we mainly focused on the concentration changes in HbO and HbR in the prefrontal cortex.

Functional near-infrared spectroscopy is also an effective approach to explore the temporal and spatial states of the human brain (Maki et al., 1995). It provides a balance between temporal and spatial resolution in comparison with other neurophysiological modalities, making it a viable option for mental workload estimation (Isbilir et al., 2019). In the present study, we focused on investigating the channel-wise analysis of fNIRS spatial features to explore the most important brain regions for predicting programming ability.

A recurrent neural network (RNN) is usually considered the best neural network structure for time series prediction, but recent studies have shown that a convolutional neural network (CNN) can perform these predictions comparably not only with greater accuracy but also more easily and clearly (Bai et al., 2018), particularly when there are many similar time series to learn from Chen et al. (2019). Dilated convolutions can make one-dimensional CNNs effectively learn time series dependencies (Yu and Koltun, 2016; Borovykh et al., 2018). In RNNs, the prediction of subsequent time steps must occur after the previous time step has been completed. In contrast, convolutions can be performed in parallel because the filters used in each layer are the same. Therefore, in training and evaluation, CNNs can process long input sequences simultaneously rather than sequentially as with RNNs (Bai et al., 2018). Since fNIRS signals in the n-back task show multiple similar time series, and we aimed to capture features over the global theoretical receptive fields, a CNN was the best choice for the backbone network in the present study. CNNs have been widely used for automatic fNIRS signal analysis (Trakoolwilaiwan et al., 2017; Janani et al., 2020) and have been used to investigate mental workload levels using multichannel fNIRS signals (Ho et al., 2019; Saadati et al., 2020). One previous study employed a CNN to analyze fNIRS features during an n-back task and proved that CNNs can learn features automatically and obtain accurate results (Yang et al., 2020).

False discovery rate (FDR) measurements (Singh and Dan, 2006) and statistical parametric mapping (SPM) (Koh et al., 2007) have been applied for channel-wise analysis for fNIRS signals. However, these statistical analysis methods corrupt the temporal domain information in fNIRS signals. Among deep neural networks, squeeze-and excitation network (SENet).

SENet, NIRSIT, PET. represents the pioneering concept of channel attention (Guo et al., 2022). The traditional pooling layer reduces the feature map, resulting in damage to channel important information. In contrast, a squeeze-and-excitation (SE) block is a type of attention layer that can collect channel important weight in train processing. The SE block can be used to collect global information, capture channel-wise relationships, and incorporate spatial attention into the structure of the CNN (Hu et al., 2020), thereby improving the interpretability of neural networks. Moreover, in comparison with the application of convolution in feature mapping, the computational cost of SE and weighted summation is very low (Guo et al., 2022). However, SENet, which is an advanced, novel channel-attention network, has not been reported for use in fNIRS signal analysis.

To the best of our knowledge, no previous study has directly explored the brain mechanisms underlying the relationship between working memory and programming ability by using fNIRS data. Thus, the first aim of the current study was to validate the relationship between programming ability and working memory by using an n-back task. The second aim was to investigate whether the fNIRS signals detected during the performance of n-back tasks can predict the participant’s programming ability. The third aim was to explore the capability of the attention-based CNN method to analyze the spatial information of the fNIRS signals to identify the optimal brain regions to predict programming ability. Thus, we aimed to explore whether general psychological experiments could be used to predict learners’ programming ability, and to provide neuroscience evidence for the findings.

## Materials and methods

### Participants

Thirty-five participants (17 novices and 18 advanced students) were recruited from the School of Information and Electronic Engineering, Zhejiang University of Science and Technology in China. The novice group included 13 male and four female participants, while the advanced group included 14 male and four female participants. All participants were over 18 years of age [mean ± standard deviation (SD), 20.61 ± 1.23 years].

The novices were freshmen from C++ courses who had not undergone programming-intensive training previously. On the other hand, the advanced students were from the programming competition team who had at least 2 years of programming-intensive training and had at least received an award in the international collegiate programming contest (We did not investigate the effect of programming training on working memory in the present study, and merely used this approach to select participants). Before the experiment, the participants were asked to complete a programming level test, which consisted of ten items with ten points for each completely correct answer and deductions for incomplete results. The maximum total score in the programming level test was 100. The advanced students had higher scores on the programming level test than the novices (mean ± SD, 83.9 ± 5.96 vs. 50.0 ± 7.71). An independent-sample *t*-test revealed that the scores on the programming level test were significantly different between the two groups [*F* (1, 34) = 214.07, *p* < 0.001,$\eta_{p}^{2}=$ 0.87].

All participants signed the informed consent form before the experiment and received a small gift at the end of the study to thank them for their time and effort.

### Experimental setup and tasks

The participants were assigned to two groups: novice and advanced students. The experimental procedures were conducted through computer programs based on E-prime, a general psychological experiment software. Each participant was individually tested in a laboratory environment for approximately 30 min. Before completing the task, participants learned about the experimental procedure. The participants were asked to relax and do nothing as the baseline task, and measurements obtained during this baseline task were used as the baseline for comparison of fNIRS signals.

**FIGURE 1:**
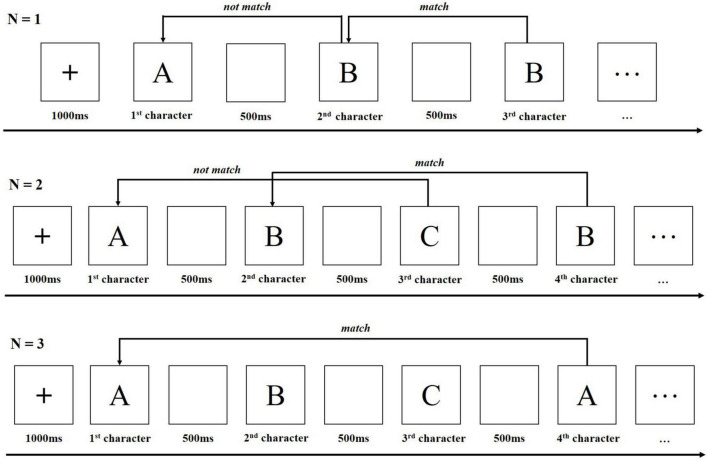
Trial schematic of the n-back task conditions.

The present study employed an n-back task to investigate the participants’ working memory. The participants monitored a series of character stimuli and responded whenever a stimulus presented was the same as the one presented n trials previously (Owen et al., 2005). The main n-back task involved 30 blocks, with 10 blocks of each n-back level presented pseudorandomly (Braver et al., 1997). Figure 1 shows the trial schematic of the n-back task conditions in the present study. For example, in the 2-back task, the third C did not match the first A. However, the fourth B matched the second B, so the participants were required respond positively whenever the character they saw was the same as the one they viewed two characters earlier. fNIRS data were recorded continuously during the entire session.

### Functional near-infrared spectroscopy data acquisition

In the present study, we focused on the HbO and HbR concentration changes in the prefrontal cortex. The hemodynamic responses measured in the prefrontal cortex were consistent enough to distinguish three levels of n-back workloads (Owen et al., 2005; Herff et al., 2013).

**FIGURE 2:**
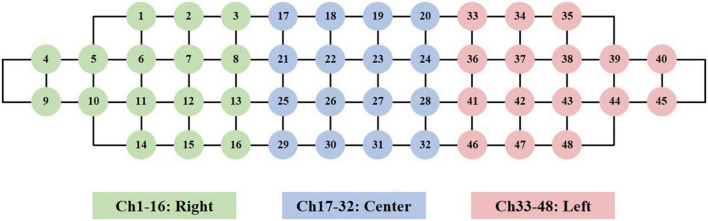
Channel configuration in our experiment.

Functional near-infrared spectroscopy (fNIRS) data were recorded at a sampling rate of 8.13 Hz using a wearable NIRS device, the NIRSIT model from OBELAB (Korea). The NIRIST device has a comprehensive 48-channel system and can capture depth-dependent hemodynamic changes in the prefrontal cortex. This system utilizes 24 laser sources (780/850 nm; maximum power under 1 mw) and 32 photodetectors (Choi et al., 2016). The grouping of NIRSIT channels is shown in Figure 2 and includes the right (#1–16), center (#17–32), and the left (#33–48) regions.

### Functional near-infrared spectroscopy data pre-processing

**FIGURE 3:**
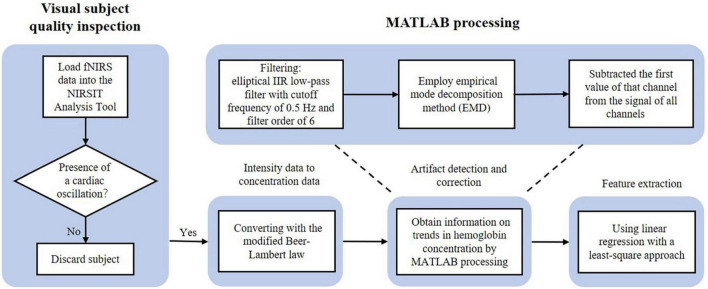
Flow diagram of functional near-infrared spectroscopy (fNIRS) data pre-processing for the study.

Figure 3 presents the flow diagram for fNIRS data pre-processing. We first loaded fNIRS data into the NIRSIT Analysis Tool for visual inspection, segmentation of the main n-back trials from practice trials, and division of the prefrontal cortex into three regions—right, center, and left—as shown in Figure 2. We then performed visual inspection at the participant level to examine overall data quality and to evaluate the quality of the data obtained from both sides of the prefrontal cortex, which showed a much lower signal-to-noise ratio (SNR) than the data from the center of the prefrontal cortex.

Since each participant had a different completion time, we tailored the data with the shortest completion time. Thus, the three n-back tasks had different data lengths. However, in the subsequent data processing, we focused on the slope and statistical features that are less affected by data length.

Visual inspection was performed by examining the spectrogram of every channel to identify the presence of cardiac oscillations, which are typically approximately 1 Hz (Tong et al., 2011). The presence of this cardiac signal is a good indicator that the optical density signals are successfully coupled with a physiological hemodynamic response (Hocke et al., 2018). This method was employed for preliminary selection of participants. In this visual inspection, one participant with unusable data, which was defined by the presence of more than seven unclean channels in one area, was identified and excluded from further analyses. Thus, the novice and advanced student groups included data from 17 participants each.

Then, we used the NIRSIT Analysis Tool to convert the raw light intensity data into HbO and HbR concentrations by means of the modified Beer–Lambert law (Sassaroli and Fantini, 2004). However, the signals still contained biological and technical artifacts. Several cardiovascular phenomena, such as heart beats, respiration, and blood pressure (Mayer waves), influenced the recorded data. Movement artifacts such as high-frequency spikes, shifts from baseline intensity, and low-frequency variations, which are present in most fNIRS datasets, can severely affect the quality of recorded data (Franceschini et al., 2006; Huppert et al., 2009).

Therefore, we conducted further data processing in Python-SciPy. To attenuate heartbeat and other biological signals, we used an elliptical Infinite Impulse Response (IIR) low-pass filter with a cutoff frequency of 0.5 Hz and a filter order of 6, which robustly removed biological artifacts in the data (Herff et al., 2013). Then, we tried to use the wavelet artifact removal method to reduce the effect of movement artifacts. Since the signals showed channel- and participant-wise variations and the wavelet basis function had limited adaptability, we found it difficult to identify a suitable wavelet basis function to remove the movement artifacts effectively. Therefore, we used the empirical mode decomposition (EMD) method, which can decompose signals without any additional parameters and therefore robustly reduced the influence of movement artifacts and Mayer wave-like effects in the data. Some of the spontaneous physiological information, such as breathing rate (∼0.3 Hz) and very low-frequency oscillations (<0.01 Hz), were still reflected in the data obtained for post-processing analysis. Therefore, we used the EMD method to deal with this noise after applying a low-pass filter, since direct application of a high-pass filter would have destroyed other useful signal components. Furthermore, due to various factors, the amplitude and intensities of the acquired hemodynamic signals differed significantly among participants. To attenuate the influence of a single participant’s data on the grand average of acquired hemodynamic signals, we subtracted the first value of that channel from the signal of all channels and rejected channels that may have significantly influenced the grand average. After these treatments, we obtained information on trends in HbO and HbR concentrations.

The slope of the trend data (Herff et al., 2013) is often used as a simple but effective feature. To obtain this slope, we fitted a straight line to the data using linear regression with a least-squares approach.

### Attention-based convolutional neural network for functional near-infrared spectroscopy spatial feature analysis

**FIGURE 4:**
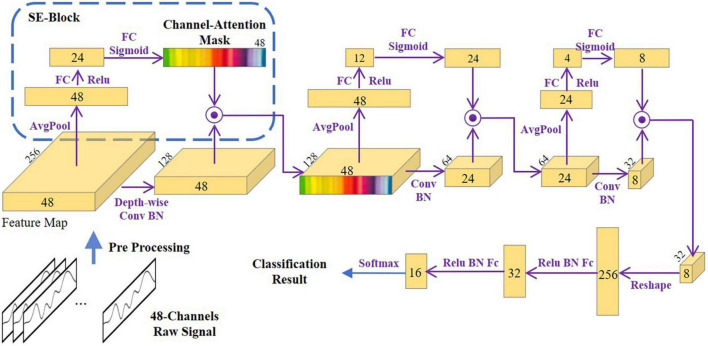
The structure of the attention-based convolutional neural network.

The structure of the attention-based CNN that we introduced to analyze the fNIRS signals is depicted in Figure 4, with the channel-attention blocks showing the spatial importance of fNIRS channels. After data pre-processing as described in Section “Functional near-infrared spectroscopy data pre-processing,” each channel of data was resampled and rescaled to a uniform length *L* = 256. Then, we stacked all channels to build a 48 × 256 feature matrix and used direct resampling and rescaling. The fNIRS signals did not contain periodic frequency information, which may have been corrupted by those processes.

In our training procedure, the mean-squared-error (MSE) method was chosen for the loss function.


$$\text{MSE}=\frac{1}{n}\,\sum_{i=1}^{n}{\left(y_{i}-{\bar{y}}_{i}\right)}^{2}$$

Here, we choose Adam as our optimizer and set the initial learning rate to 0.01.

Three SE blocks were inserted into three normal convolution layers. After the global average pooling operator, each channel data point was consolidated into one data point. In the first SE block, the fully connected layer transforms the 1 × 48 vector to 1 × 24; this process is also called the “squeeze.” The squeezing function also serves to embed the global distribution of feature responses over all channels. This operator is followed by an excitation operator, which consists of a fully connected layer and a sigmoid activation layer. Excitation is a self-gating mechanism (Hu et al., 2020) that produces a mask representing the per-channel modulation weights. These weights are then applied to the original feature map to generate the new output. This series operation is also known as the self-attention operation (Vaswani et al., 2017). The feature vector needs to be squeezed small and then return to the origin scale *via* excitation because we aimed to improve the training pressure and to prevent overfitting. The reduction factor needs to be carefully adjusted within the training process. A special classified task was chosen. After proper training, we opened the SE block to observe the channel-wise weights mask.

The backbone network of the attention-based CNN we used (as shown in Figure 4) had a traditional CNN structure. Here, FC refers to the fully connected layer; BN refers to the batch-normalization layer; and the two round circles indicate the dot-product operator. However, to prevent mixing of the channel information, a generic convolution (GC) layer cannot be used at the beginning of the network. As illustrated in Figure 4, the key point is to replace the first GC layer with a depth-wise convolution (DC) layer before the self-attention mechanism finds the important channels. Nevertheless, the other convolution layers are still GC layers. The DC layer ignores the interchannel information, which must be remedied with a point-wise convolution (PC) layer. This will complicate the overall structure of our neural network.

Due to the limited amount of training data in this study, the neural network was easily overfitted. However, in our training procedure, we were not overtly concerned with the generalization properties of the neural network. Instead, we aimed to reveal the importance of channels. The main purpose of this model is to train the channel-attention block, and some degree of overfitting can help make important channels more obvious (Hu et al., 2020). Thus, model overfitting can be acceptable for general inference.

## Results

The criterion for statistical significance was set at *p* < 0.05. The Greenhouse–Geisser correction was used to compensate for sphericity violations. Effect sizes were measured by $\eta_{p}^{2}$, with $\eta_{p}^{2}$ = 0.01, 0.06, and 0.14 indicating small, medium, and large effects, respectively (Fritz et al., 2012).

### n-back performance

The descriptive statistics for accuracy and reaction time in each group are presented in Table 1. Accuracy was calculated by determining the average percentage of correct trials under each back condition, while reaction time was computed by determining the mean across correct trials for each back condition. As shown in Table 1, novices performed the trials with lower accuracy and slower reaction times than advanced students over each back level.

**TABLE 1:** Means and standard deviations of accuracy and reaction time during the n-back task.

Dependent variable	Accuracy				Reaction time (ms)			
	Novices		Advanced students		Novices		Advanced students	
	M	SD	M	SD	M	SD	M	SD
1-back	0.986	0.023	0.992	0.019	550	113	474	53.2
2-back	0.983	0.033	0.986	0.032	751	152	654	75.5
3-back	0.845	0.080	0.931	0.065	1276	222	1087	196

Paired *t*-tests indicated that the accuracies for the 1-back and 2-back conditions were near the ceiling levels and did not differ in both advanced students and novices. Accuracy for the 3-back condition was marginally significantly lower than those for the 1-back [*t* (16) = 1.984, *p* = 0.073] and the 2-back [*t* (16) = 2.072, *p* = 0.063] conditions among the advanced students. However, accuracy for the 3-back condition was significantly lower than those for the 1-back [*t* (16) = 4.690, *p* = 0.001] and the 2-back [*t* (16) = 3.801, *p* = 0.003] conditions among the novices.

Paired *t*-tests indicated that the 1-back task was performed faster than the 2-back [*t* (16) = −4.641, *p* = 0.001; *t* (16) = −4.935, *p* < 0.001] and 3-back [*t* (16) = −8.567, *p* < 0.001; *t* (16) = −11.950, *p* < 0.001] tasks by the advanced students and the novices, respectively, while the 2-back task was performed faster than the 3-back [*t* (16) = −7.637, *p* < 0.001; *t* (16) = −8.368, *p* < 0.001] task by both advanced students and the novices.

To examine group differences, we conducted one-way repeated-measures analysis of variance (ANOVA) with programming ability (novices vs. advanced students) as the between-subjects factor and n-back levels (1-back, 2-back, and 3-back) as the within-subject factor.

One-way repeated-measures ANOVA revealed a marginally significant main effect of programming ability on accuracy [*F* (1, 32) = 3.867, *p* = 0.075, $\eta_{p}^{2}=$ 0.260]. The interaction between programming ability and the n-back task was not significant [*F* (2, 64) = 1.692, *p* = 0.207, $\eta_{p}^{2}=$ 0.133].

One-way repeated-measures ANOVA with Greenhouse–Geisser correction revealed a main effect of programming ability on reaction time [*F* (1, 32) = 5.650, *p* = 0.029, $\eta_{p}^{2}=$ 0.239]. The interaction between programming ability and the n-back task was also not significant [*F* (2, 64) = 1.177, *p* = 0.304, $\eta_{p}^{2}$ = 0.061].

**FIGURE 5:**
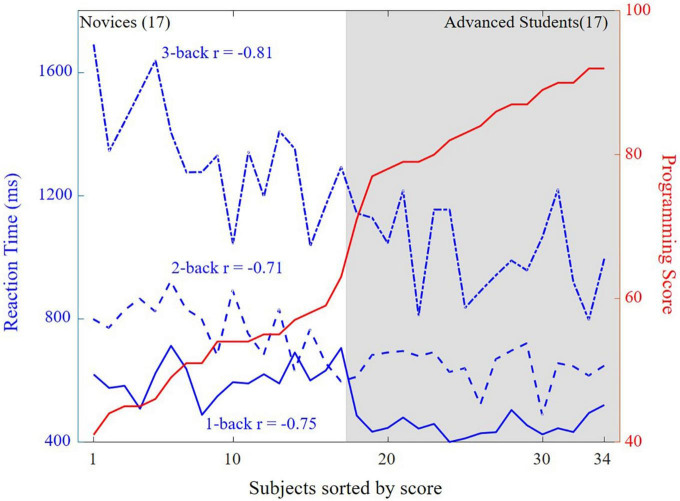
Correlations between reaction time and programming score for the three n-back levels.

Figure 5 illustrated that the correlations between reaction time and programming score were negative for 1-, 2-, and 3-back levels (*r* = −0.75, *r* = −0.71, and *r* = −0.81), i.e., the reaction time was faster for a higher programming score.

### Functional near-infrared spectroscopy hemodynamic responses

To determine the differences in hemodynamic responses between novices and advanced students under the three n-back conditions, we first analyzed the grand averages of all participants.

**FIGURE 6:**
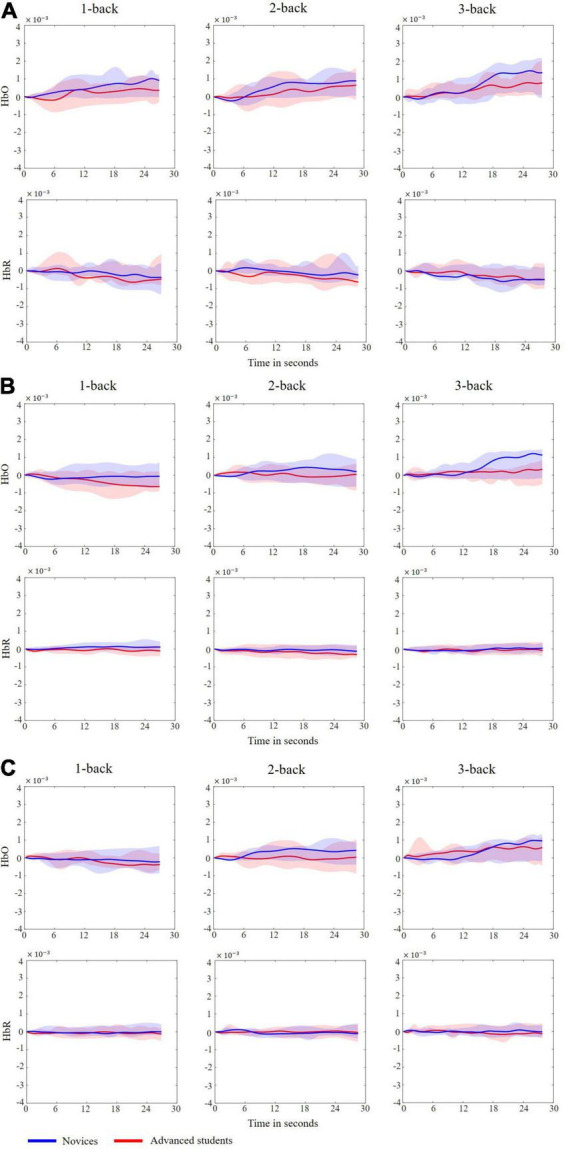
Grand averages of all participants in the three n-back levels. **(A)** Left prefrontal cortex. **(B)** Center prefrontal cortex. **(C)** Right prefrontal cortex.

Figure 6 exhibited the grand averages of all participants for the three n-back levels. The blue lines showed the grand averages for novices, while the magenta line showed the overall mean for advanced students. For HbO, a clear increase was observed at the 1-, 2-, and 3-back levels, and the slope was positive for all three n-back conditions in the left, center, and right prefrontal cortices. The grand average increase was steeper in the 2-back task than in the 1-back task and was the steepest in the 3-back task.

For HbR, a slight decrease in concentration changes can be seen for all three n-back conditions, and the slope was negative for the three n-back levels; conversely, there was no obvious difference between the 1- and 2-back grand averages and the 3-back grand average.

One-way repeated-measures ANOVA revealed a main effect of programming ability on HbO [*F* (1, 32) = 8.838, *p* = 0.007, $\eta_{p}^{2}$= 0.287; *F* (1, 32) = 12.713, *p* = 0.002, $\eta_{p}^{2}$= 0.366; *F* (1, 32) = 25.805, *p* < 0.001, $\eta_{p}^{2}$= 0.540] in the right, center, and left prefrontal cortices. The interaction between programming ability and the n-back task was not significant.

**FIGURE 7:**
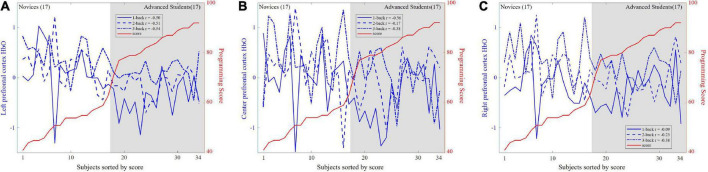
Correlations between hemoglobin (HbO) and programming score for the three n-back levels in the left, center, and right prefrontal cortices. **(A)** Left prefrontal cortex. **(B)** Center prefrontal cortex. **(C)** Right prefrontal cortex.

Figure 7 illustrated that the correlations between HbO and programming score were negative for the three n-back levels in the left (*r* = −0.50, −0.51, −0.54), center (*r* = −0.36, −0.17, −0.38), and right (*r* = −0.09, *r* = −0.23, *r* = −0.38) prefrontal cortices, i.e., HbO is lower for higher programming scores.

One-way repeated-measures ANOVA revealed that the main effect of programming ability on HbR [*F* (1, 32) = 0.001, *p* = 0.980, $\eta_{p}^{2}$< 0.001; *F* (1, 32) = 2.716, *p* = 0.114, $\eta_{p}^{2}$ = 0.110; *F* (1, 32) = 0.104, *p* = 0.750, $\eta_{p}^{2}$= 0.005] was not significant in the right, center, or left prefrontal cortices, and there was no interaction between programming ability and the n-back task.

The results indicated that HbO can indicate working-memory load and show significant associations between brain activity and programming ability, but HbR cannot.

### Functional near-infrared spectroscopy feature analysis using attention-based convolutional neural network

To obtain the most important channels in the fNIRS signals, we constructed a virtual classification task, and tried letting the neural network model illustrate the importance of the fNIRS channels through the virtual training task. Under this virtual task, we directly combined all subject data into a signal batch and used MSEloss to train the network. After approximately 50 epochs, the loss stopped falling, and we obtained almost 100% accuracy. Because the amount of data was not very large, the generalization ability of the network was weak. However, we were not going to use this trained network for general classification in a new set of data. We only needed to observe the network’s understanding of channel importance under this task. In the present study, we used the mean value of 10 training sessions for subsequent analysis.

**FIGURE 8:**
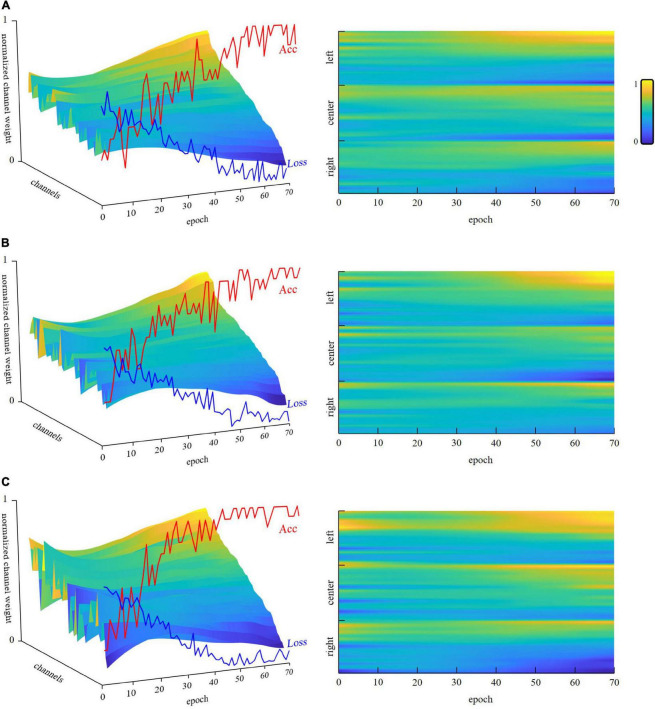
The channel weights fitting with the training process of the attention-based convolutional neural network (CNN). The left panel shows all channel weights sorted in the order of the final training results. All channels are sorted by the last loss value. The right panel presents a top-down view of the left panel. However, the channels are re-ranked into the left, center, and right regions. We list the three groups in Supplementary Table 1. The right panel has two axes: re-ranked channels and epochs. The normalized channel weight is represented by color bars. **(A)** 1-back, **(B)** 2-back, **(C)** 3-back.

The left panels in Figure 8 showed the channel weights fitting with the training process. The left panel showed that the accuracy (red curve) was close to 100% with the loss (blue curve) down to zero. Furthermore, we distinguished those weights into three brain regions (see Figure 2), as shown in the right panels. After fitting, the left prefrontal cortex showed an obviously high weight in the three n-back levels. These findings were consistent with the results of one-way repeated-measures ANOVA, in which the left prefrontal cortex had a larger effect size than the right and center prefrontal cortices.

## Discussion

The main purpose of the present study was to investigate the brain mechanisms underlying the relationship between working memory and programming ability by using fNIRS signals.

The analysis of participants’ n-back performance showed differences in the accuracy and reaction time depending on the n-back level between novices and advanced students. Advanced students performed better than novices in terms of both accuracy and reaction time. Since the n-back task is recognized as an effective method to measure working memory, a better n-back performance indicated higher working-memory capacity (Kirchner, 1958). The study results validated the relationship between programming ability and working memory, and students with higher working-memory capacity showed better programming ability. The results also provided evidence that limited working-memory capacity has negative effects on learning (Alloway, 2009).

Since the n-back task may be easier for advanced students, the advanced participants were expected to show less prefrontal cortex activation during each n-back experiment (Asgher et al., 2019; Khoe et al., 2020). Figures 6, 7 illustrated the neural evidence of this finding. The hemodynamic responses of HbO associated with n-back stimulus presentation increased more in novices than in advanced students. The results of the statistical analyses revealed that HbO in the prefrontal cortex showed significant differences between novices and advanced students during the n-back task. Thus, HbO signals measured during the n-back test can be used to robustly predict the programming ability of students. The changes in cerebral blood oxygen signals represent the changes in local oxygen consumption caused by local brain activity and reflect the activity state of the human brain (Strangman et al., 2002). According to the neural efficiency hypothesis (Haier et al., 1988), the higher the performance in related fields (the higher the cognitive ability), the lower the activation degree of the cerebral cortex, showing a negative correlation. Thus, in comparison with a lower cognitive ability group, a higher cognitive ability group shows lower activation of brain regions when performing tasks with the same difficulty (Dunst et al., 2014; Genc et al., 2018). A higher working-memory load tends to produce greater prefrontal cortex activation (Herff et al., 2013). The novices exhibited significantly higher HbO concentration increments than their advanced counterparts during the n-back tests. Thus, the working-memory load in novices was higher and consumed more mental resources. In comparison with the novices, the advanced students illustrated lower prefrontal cortex activation for the n-back task, which was considered to place less of a demand on working-memory load and was easier to complete for the advanced students. This is the underlying basis for the measurement of prefrontal cortex activation of fNIRS signals during n-back tasks to predict an individual’s programming ability.

The results also indicated the possibility of predicting students’ programming potential through general psychological experiments such as n-back test (as shown in Figure 5). This method may be especially useful for evaluating individuals with no programming foundation.

Additionally, HbR activation reduced slightly during the n-back task, as illustrated in Figure 6, and programming ability showed no main effect on HbR. This may be because relative to HbO, the HbR concentration is weak and difficult to detect in real time (Abibullaev and An, 2012), making it harder to detect significant effects on HbR activation in task-based fNIRS (Huppert et al., 2006).

Although the main effects of programming ability on HbO were significantly different in the left, middle, and right prefrontal cortices, by applying the deep learning method of attention-based CNN to the fNIRS signals, we found that the channels that can better distinguish programming ability were in the left region of the prefrontal cortex (see Figure 8). CNNs can extract microfeatures from temporal domain signals that may be corrupted by statistical analysis. CNNs can also be used to discover and extract the appropriate internal structure through convolution and pooling operations and automatically generate the deep features of the raw data. Moreover, the deep features are robust against translation and scaling (Zhao et al., 2017); they work well in discarding noisy series and can extract meaningful patterns while ignoring patterns without value (Aussem and Murtagh, 1997). By introducing attention modules (i.e., the SE block), we can open the black box to see which feature the CNN network relies on to identify those signals. As the CNN network is gradually fitted, the attention modules indicate the important channels, as shown in Figure 8. These high-weight channels are those that the neural network uses to understand and classify. In other words, these channels and their corresponding brain regions have higher resolution in this task. The SE block can also improve the representational power of the regular CNN by offering it a kind of dynamic channel-wise fixing feature (Hu et al., 2020). Furthermore, the feature importance values produced by the self-attention operation can be used for model pruning, which can lead to the construction of more efficient physiological signal analysis networks.

The results of the current study also provided further evidence to support the lateralization of brain functions. The left prefrontal cortex was more important in programming ability prediction, as demonstrated in Figures 7, 8. Many studies have reported functional hemispheric asymmetry in cognitive processes (Gazzaniga, 1989, 1995; Goel, 2019). Smith et al. (1996) and Smith and Jonides (1997) used PET technology to study the neural basis of working memory with the n-back paradigm. Their results showed that the activation areas in the verbal and spatial n-back tasks are different: the former activates the left hemisphere, and the latter activates the right hemisphere. Baddeley and Logie (1999) also reviewed the evidence showing that the left hemisphere is associated with verbal working-memory tasks. As shown in Figure 6, our results also demonstrated that left prefrontal cortex regional activation was more dynamic during the verbal n-back test.

This study had some limitations that should be addressed in future studies. First, we did not consider the mediating factors between working memory and programming ability. Previous studies have shown that the relationship between working memory and academic performance is mediated by visuospatial abilities (Logie et al., 2000) and the ability to control attention (Kane and Engle, 2003). Future studies should aim to control these mediating factors to acquire more rigorous results. Second, the number of participants who met the criteria for advanced students was relatively small. Further studies with additional data are required to improve the generalizability of the findings.

## Conclusion

To the best of our knowledge, few empirical studies have directly examined the relationship between working-memory capacity and programming ability, and no studies have provided direct neural evidence to support this relationship. The present study attempts to fill this gap and demonstrates that students’ programming ability can be predicted by evaluation of their working-memory capacity while providing direct neural evidence supporting this relationship. The results of our analyses indicate that fNIRS detected functional neural changes associated with the workload in the prefrontal cortex, demonstrating that the hemodynamic responses measured in the prefrontal cortex can be used to discriminate between novices and advanced students. Additionally, we utilized an attention-based CNN to analyze the spatial domains of the fNIRS signals and demonstrated that the left prefrontal cortex was more important than other brain regions for programming ability prediction.

## Data availability statement

The datasets presented in this study can be found in online repositories. The names of the repository/repositories and accession number(s) can be found below: https://github.com/GiantMushroom/NIRdataset.git.

## Ethics statement

The studies involving human participants were reviewed and approved by the Ethics Committee of the School of Information and Electronic Engineering, Zhejiang University of Science and Technology. The participants provided their written informed consent to participate in this study.

## Author contributions

XG: methodology and writing – original draft preparation. YL: conceptualization, validation, writing – review and editing, and funding acquisition. YZ: formal analysis and writing – original draft preparation. CW: investigation and data curation. All authors contributed to the article and approved the submitted version.
