# Supplementary material for: Programming ability prediction: Applying an attention-based convolutional neural network to functional near-infrared spectroscopy analyses of working memory
Source: Front Neurosci. 2022 Dec 1;16:1058609. doi: 10.3389/fnins.2022.1058609 (PMC9751487; doi:10.3389/fnins.2022.1058609)
Supplement: Supplementary file 1 [file Table_1.DOCX]

**Appendix table 1: Channels’ rank corresponding to three brain regions**

|  | 1-back | 2-back | 3-back |  | 1-back | 2-back | 3-back |  | 1-back | 2-back | 3-back |
| --- | --- | --- | --- | --- | --- | --- | --- | --- | --- | --- | --- |
| left | 36 | 36 | 44 | Center | 31 | 26 | 23 | Right | 6 | 1 | 6 |
|  | 35 | 33 | 35 |  | 27 | 23 | 20 |  | 2 | 14 | 9 |
|  | 47 | 37 | 34 |  | 21 | 28 | 29 |  | 14 | 2 | 16 |
|  | 44 | 47 | 37 |  | 24 | 29 | 25 |  | 12 | 15 | 8 |
|  | 41 | 40 | 42 |  | 20 | 18 | 19 |  | 8 | 9 | 12 |
|  | 46 | 45 | 41 |  | 19 | 27 | 26 |  | 15 | 16 | 7 |
|  | 33 | 48 | 47 |  | 26 | 25 | 30 |  | 7 | 7 | 11 |
|  | 45 | 46 | 36 |  | 17 | 19 | 31 |  | 4 | 10 | 5 |
|  | 39 | 43 | 45 |  | 25 | 24 | 27 |  | 9 | 6 | 4 |
|  | 48 | 42 | 33 |  | 22 | 31 | 28 |  | 10 | 11 | 14 |
|  | 40 | 41 | 40 |  | 29 | 17 | 18 |  | 3 | 4 | 15 |
|  | 42 | 35 | 43 |  | 28 | 21 | 32 |  | 16 | 3 | 13 |
|  | 34 | 39 | 48 |  | 32 | 30 | 21 |  | 11 | 13 | 10 |
|  | 38 | 44 | 39 |  | 23 | 32 | 17 |  | 1 | 5 | 3 |
|  | 43 | 34 | 38 |  | 18 | 20 | 24 |  | 13 | 8 | 2 |
|  | 37 | 38 | 46 |  | 30 | 22 | 22 |  | 5 | 12 | 1 |
